# Dynamic constitutive identification of concrete based on improved dung beetle algorithm to optimize long short-term memory model

**DOI:** 10.1038/s41598-024-56960-z

**Published:** 2024-03-15

**Authors:** Ping Li, Haonan Zhao, Jiming Gu, Shiwei Duan

**Affiliations:** 1 School of Management Science and Engineering, Anhui University of Technology, Ma’anshan, 243032 China; 2 School of Mechanical Engineering, Anhui University of Technology, Ma’anshan, 243032 China

**Keywords:** Dynamic constitutive model of concrete, Dung beetle optimization algorithm, Long short-term memory network, PID control, Lens imaging reverse learning, Engineering, Materials science

## Abstract

In order to improve the accuracy of concrete dynamic principal identification, a concrete dynamic principal identification model based on Improved Dung Beetle Algorithm (IDBO) optimized Long Short-Term Memory (LSTM) network is proposed. Firstly, the apparent stress–strain curves of concrete containing damage evolution were measured by Split Hopkinson Pressure Bar (SHPB) test to decouple and separate the damage and rheology, and this system was modeled by using LSTM network. Secondly, for the problem of low convergence accuracy and easy to fall into local optimum of Dung Beetle Algorithm (DBO), the greedy lens imaging reverse learning initialization population strategy, the embedded curve adaptive weighting factor and the PID control optimal solution perturbation strategy are introduced, and the superiority of IDBO algorithm is proved through the comparison of optimization test with DBO, Harris Hawk Optimization Algorithm, Gray Wolf Algorithm, and Fruit Fly Algorithm and the combination of LSTM is built to construct the IDBO-LSTM dynamic homeostasis identification model. The final results show that the IDBO-LSTM model can recognize the concrete material damage without considering the damage; in the case of considering the damage, the IDBO-LSTM prediction curves basically match the SHPB test curves, which proves the feasibility and excellence of the proposed method.

## Introduction

Concrete is one of the most common building materials in civil engineering, and it is the basis for building engineering to be realized. Its quality and performance will have a direct determining effect on the quality of engineering projects. During the service of engineering structures, they not only bear quasi-static loads, but also withstand shocks or explosions due to accidental accidents, such as gas explosions, terrorist attacks and explosions caused by improper operation in factories, resulting in high strain rate and high temperature environment of concrete materials in construction projects, resulting in a large number of casualties. The dynamic failure of materials is actually a process of nucleation-growing-connectivity of cross-scale cracks in materials (possibly accompanied by the closure of a few cracks), and the evolution rate of cracks is a function of time/loading rate^1^. At present, the study of dynamic damage of brittle concrete materials has always been a hot and difficult problem for scholars at home and abroad. The traditional concrete dynamic constitutive model is a mathematical equation in explicit function form that can reflect the performance of concrete material to a certain extent by means of macroscopic or microscopic experimental techniques and based on continuum mechanics or microscopic damage mechanics. Such as: Holmquist-Johnson–Cook (HJC) model^2^, Taylar–Chen–Kuszmaul (TCK) model^3^, Z–W–T model^4^ and so on. In the process of establishing the dynamic constitutive model, the factors such as loading form, loading rate, temperature and damage as well as the coupling between these factors should be taken into account. Because the material parameters are difficult to determine accurately, and even some parameters (such as damage) can not be directly measured, and solving the mathematical equation is too complicated and lacks practicability. Therefore, at present, there is no constitutive model which is convenient for engineering application and can accurately and comprehensively describe the factors affecting the performance of concrete materials and the coupling effects between the factors.

With the development of artificial intelligence, machine learning (ML), especially deep learning (DL) algorithm, has been widely used in damage detection, system identification and risk assessment of engineering structures^5^. The application of machine learning to concrete damage identification and detection can be roughly divided into two categories. One is to collect images of cracks and interface damage in concrete structures, and combine them with deep learning to detect them. For example, Song et al.^6^, based on the electric drive platform, proposed a method of close-range scanning to capture high-resolution panoramic images of the surface of concrete structures, adopted convolutional neural network to automatically segment panoramic cracks, and quantified panoramic cracks through crack matching and performance calculation methods. Laxman et al.^7^ developed an integrated CNN model based on the binary class convolutional neural network (CNN) model and combined the convolutional feature extraction layer with the regression model (RF and XGBoost) to automatically detect cracks on the concrete surface and evaluate crack depth. Jiang et al.^8^ used deep separable convolution, inverse residual network and linear bottleneck structure to optimize the original YOLO-v3 and SSD target detection algorithms, and the complete detection accuracy rate reached about 65%. Cui et al.^9^ improved YOLO-v4 by combining converter theory and proposed an MHSA-YOLOv4 target detection algorithm adapted to concrete wind erosion damage. The results show that the improved algorithm can accurately identify wind erosion damage in concrete images. The width and depth of concrete cracks can be determined by analyzing concrete images containing damage and combining with deep learning algorithm, but this method can only randomly detect the results of local crack evolution of concrete, but can not get the process of concrete crack evolution. The other is to treat the material damage identification process as a system identification problem. It is therefore possible to determine a system model that identifies the relationship between cause (input) and effect (output).

For example, Sun^4^, Xu et al.^10^ studied the dynamic constitutive model of polymers at high strain rates by combining SHPB technology with BP neural networks, with or without damage evolution. Sun et al.^11^ defined the damage of concrete as the deterioration of compressive strength and tensile strength according to the continuum damage mechanics theory. RBF neural network was used to model the damage of concrete under different freeze–thaw cycles, and the number of freeze–thaw cycles, dynamic elastic modulus loss, mass loss and stress ratio were taken as input variables. Concrete damage value as output variable. It is a new way to study the dynamic mechanical properties of concrete from the perspective of system science. The constitutive response and damage evolution law of concrete under different impact loads can be identified by the constructed model according to the test data without any constitutive assumptions in advance. At present, the concrete dynamic constitutive identification model is only a single model, such as BP neural network and RBF neural network, which has the advantages of parallel processing and self-learning, but the single algorithm model is too simple in operation and the identification error is large.

Long-term Short-Term Memory (LSTM)^12^ is a variant of Recurrent Neural networks (RNN). Mainly to solve the problem of gradient disappearance and gradient explosion in the process of long sequence training, LSTM is specially used for processing sequence data. Compared with traditional RNN structure, LSTM is time-sensitive and can learn patterns and features in time series data. A gating mechanism is introduced to better capture long-term dependencies in sequence data. Zhou et al.^13^ used LSTM models (LSTM, Bi-LSTM, Dense-LSTM) for time series prediction, and compared the experimental results with each other, indicating that LSTM model is one of the most advanced models for predicting time series data. Therefore, LSTM is introduced as the main model in this paper, but since the prediction accuracy of LSTM is closely related to the selection of key parameters, the selection of appropriate parameters is the key to improve the identification of concrete dynamic constitutive. Generally, empirical values alone are not enough to meet the prediction error requirements. Geng et al.^14^ propose an improved adaptive particle swarm optimization (IAPSO) model to optimize LSTM neural network's hyperparameters (such as time step, hidden unit, batch size and period). And applied to food safety risk analysis and early warning. Li et al.^15^ proposed a model to optimize LSTM neural network based on variatory mode decomposition (VMD) and improved Dung Beetle Algorithm (IDBO), and applied it to fault detection of PV array, aiming at the problems of low convergence accuracy of Dung Beetle algorithm (DBO) and easy to fall into local optimal. Levy flight strategy, T-distribution perturbation strategy and multi-population mechanism were integrated to improve DBO. In the iterative process, Levy flight strategy was used to perturb elite dung beetles and generate candidate solutions, and combined with greedy selection and elimination of poorly fit dung beetles, the local optimization ability of the algorithm was enhanced. In the later stage of iteration, the T-distribution disturbance mechanism was used to expand the search range of dung beetles, and the predation strategy of grey Wolf algorithm was combined to promote the diversity of the population and avoid falling into local extreme values, which greatly improved the global optimization ability of dung beetle optimization algorithm.

Dung Beetle Optimizer (DBO) is an intelligent optimization algorithm proposed by Bo Shen et al.^16^ in 2022. The algorithm contains few parameters, has no special requirements for the initial setting of model parameters, and has strong universality. When dealing with complex high-dimensional optimization problems, it has the characteristics of high convergence speed, high precision and high stability. In practical application, dung beetle optimization algorithm, like other intelligent optimization algorithms, has the defect that it is easy to fall into local optimal in the late optimization period. Therefore, in view of the above problems, some scholars have improved it and applied it to different projects. Zhu et al.^17^ proposed a dung beetle optimization algorithm based on quantum computing and multi-strategy mixing, and introduced the best-point set strategy to initialize the population, so that the initial population distribution was more uniform. The T-distribution variation strategy based on quantum computing is used to change the global optimal solution and prevent the algorithm from falling into the local optimal. Zhou et al.^18^ introduced a periodic mutation mechanism into the Dung Beetle optimization algorithm to improve the optimization ability of the algorithm, and used the improved algorithm to optimize the differential integrated moving average autoregressive model to achieve the prediction of transformer vibration signals. Li et al.^19^ combined the chaotic initialization method with the reverse learning initial strategy to initialize the population and increase the diversity of the population. By integrating adaptive step size and convex lens imaging strategy and introducing random difference change strategy, the relationship between search diversity and convergence accuracy of the algorithm is balanced, and the convergence speed of DBO is improved.

Based on the above analysis, an improved Dung Beetle Algorithm (IDBO) optimized Long short-term memory (LSTM) neural network dynamic constitutive identification model of concrete is proposed in this paper. First, the apparent stress–strain curve of concrete containing damage evolution was measured by split Hopkinson pressure bar test, the damage and rheology were decouple, and the system was modeled by LSTM neural network. Secondly, based on the principle of dung beetle algorithm, improved strategies (greedy lens imaging reverse learning initializing population strategy, curve adaptive weight factor and PID control optimal solution perturbation strategy) are introduced, and their optimization performance is verified by CEC2005 test set. Finally, four LSTM hyperparameters (number of hidden units, maximum training period, initial learning rate and L2 regularization parameter) are optimized using the improved Dung Beetle algorithm. The results show that the proposed IDBO algorithm has good optimization effect and the IDBO-LSTM model has high precision in identifying concrete dynamic constitutive.

## Algorithm principle and improvement

### Dung Beetle optimization algorithm

Dung Beetle Optimizer (DBO) was inspired by dung beetle behaviors such as ball rolling, dancing, foraging, stealing and reproduction^16^. Thus, the dung beetle population consists of rolling dung beetles, breeding dung beetles, young dung beetles, and stealing dung beetles.

#### Rolling dung Beetle

In nature, dung beetles' habit is to shape animal dung into balls, which is conducive to fast and efficient movement of dung and prevent its peers from robbing them. Dung beetles need to navigate through celestial cues (sun orientation, polarized light, etc.) as they roll to keep the dung ball rolling on a straight path. As it rolls, the beetle's position updates as,

1 $$ \begin{aligned} & x_{i} (t + 1) = x_{i} (t) + \alpha \times k \times x_{i} (t - 1) + b \times \Delta x \\ & \Delta x = |x_{i} (t) - X^{\omega } | \\ \end{aligned} $$

In the formula, *t* represents the current number of iterations, $x_{i} (t)$ is where the i beetle is in the t iteration. $\alpha$ is the natural coefficient and is assigned − 1 or 1 using algorithm 1. $k \in (0,0.2]$ stands for the perturbation coefficient, set to 0.1 for a fixed value. Set the value of $b \in (0,1)$ to 0.3,$X^{\omega }$ represents the worst position in the world, $\Delta x$ is used to simulate the change of strong light.

When dung beetles hit an obstacle, they usually climb onto the dung ball and "dance" (a series of spins and pauses) to reorient themselves and gain a new route. So the beetle's position update is,

2 $$ x_{i} (t + 1) = x_{i} (t) + \tan \theta \left| {x_{i} (t) - x_{t} (t - 1)} \right| $$

In the formula, $\theta \in [0,\pi ]$ is the interference Angle, when $\theta = 0,\pi /2,\pi$, the beetle's position does not update. $\left| {x_{i} (t) - x_{i} (t - 1)} \right|$ is the difference between where the *i* beetle was on the *t* iteration and where it was on the *t* − 1 iteration.

**Algorithm 1:**
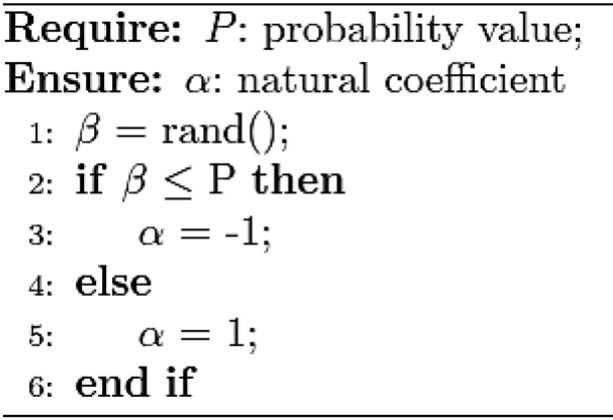
$$\alpha$$ selection strategy

#### Breeding dung beetles

In order to provide a safe environment for their young, dung beetles roll their balls to a safe place to hide. Therefore, the female dung beetle's spawning zone boundary strategy is expressed as:

3 $$ \begin{aligned} & Lb^{*} = \max (X^{*} \times (1 - R),Lb) \\ & Ub^{*} = \min (X^{*} \times (1 + R),Ub) \\ \end{aligned} $$

In the formula, $X^{*}$ represents the current local best position, $LB^{*}$ and $Ub^{*}$ are the lower and upper boundaries of the spawning area, respectively. $R = 1 - t/T_{\max }$,$T_{\max }$ is the maximum number of iterations, $Lb$ and $Ub$ represent the lower and upper bounds of the optimization problem, respectively.

After identifying the spawning area, the female dung beetle chooses that area for laying eggs. Formula (3) represents the dynamic spawning area, therefore, the position update of the oosphere in the iteration process is dynamic, which is expressed as,

4 $$ B_{i} (t + 1) = X^{*} + b_{1} \times (B_{i} (t) - Lb^{*} ) + b_{2} \times (B_{i} (t) - Ub^{*} ) $$

In the formula, $B_{i} (t)$ is the position of the *i* egg ball at the *t* iteration, $b_{1}$ and $b_{2}$ represent two independent random variables of $1 \times D$, $D$ represents the dimension of the optimization problem. Therefore, the position of the oocytes is strictly controlled within a certain range. Breeding dung beetle position update Algorithm 2 shows.

**Algorithm 2:**
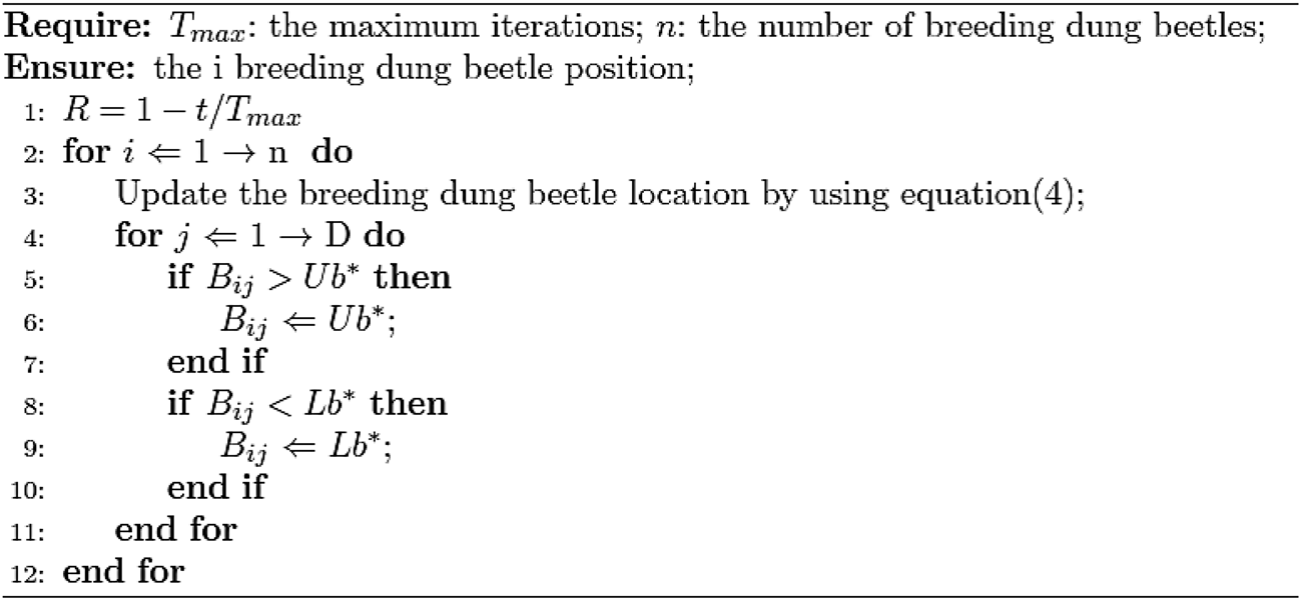
Breeding position renewal strategy of dung beetles

#### Little dung Beetle

When young dung beetles grow up after hatching eggs, they need to establish an optimal feeding area to guide them to be fed. The optimal feeding area boundary strategy is expressed as,

5 $$ \begin{aligned} & Lb^{b} = \max (X^{b} \times (1 - R),Lb) \\ & Ub^{b} = \min (X^{b} \times (1 + R),Ub) \\ \end{aligned} $$

In the formula, $X^{b}$ represents the global optimal feeding position, $Lb^{b}$ and $Ub^{b}$ are the lower and upper bounds of the optimal feeding region, respectively.

The beetle's position update is represented by,

6 $$ x_{i} (t + 1) = x_{i} (t) + C_{1} \times (x_{i} (t) - Lb^{b} ) + C_{2} \times (x_{i} (t) - Ub^{b} ) $$

In the formula,$x_{i} (t)$ is where the *i* beetle is on the *t* iteration,$C_{1}$ is a random number with a normal distribution, $C_{2} \in (0,1)$ is a random vector.

#### Dung Beetle stealing

There are some misbehaving dung beetles (thieving dung beetles) in the dung beetle population, stealing the fruits of other people's labor. According to formula (5), it is the optimal feeding position, so the setting is the best place for dung beetles to compete for food. During the iteration, the position of the thieving beetle is updated to,

7 $$ x_{i} (t + 1) = X^{b} + S \times g \times (\left| {x_{i} (t) - X^{*} } \right| + \left| {x_{i} (t) - X^{b} } \right|) $$

In the formula, $x_{i} (t)$ is where the *i* beetle is on iteration *t*, $g$ is a random vector of $1 \times D$-dimension size that follows a normal distribution, $S$ is constant.

### Improved dung beetle optimization algorithm

In the optimization problem, Dung Beetle optimization algorithm (DBO) has the advantages of high precision, faster convergence and stronger stability. According to the No Free Lunch theorem^20^, no algorithm can perform optimally in any domain. Therefore, three DBO strengthening strategies are proposed in this section to accelerate the convergence speed and enhance the global search capability of the algorithm. IDBO enhances the optimization ability of DBO through greedy lens imaging reverse learning, fusion PID control optimal solution perturbation strategy and introduction of curve adaptive factors. The pseudo-code for the improved dung beetle optimization algorithm is shown in Algorithm 3.

#### Greedy lens imaging reverse learning to initialize the population

Ideally, a good algorithm should have the final optimal solution independent of the initial position, but for almost all random algorithms, the reality is the opposite, if the initial solution is established at the optimal position in the population, the probability of the population convergence to the optimal position is very high, and determines the convergence speed and accuracy of the future algorithm^21^.Therefore, based on the uniform random initialization of the population, the introduction of lens imaging reverse learning to generate new populations, and the use of greedy idea to screen new populations from the combined population according to the fitness value, is conducive to reducing the optimization time in the algorithm iteration process. The lens imaging reverse learning strategy is mathematically expressed as,

8 $$ x_{j} (i + 1) = \frac{{ub_{j} + lb_{j} }}{2} + \frac{{ub_{j} + lb_{j} }}{2k} - \frac{{x_{j} (i)}}{k} $$

In the formula, $x_{j} (i)$ is the *i*th individual of dimension *j*, $ub_{j}$ and $lb_{j}$ are the J-dimensional components of the upper and lower bounds of the decision variable, respectively. *k* is the scaling factor.

#### PID control optimal solution perturbation strategy

In the process of iteration, the individuals in the initial population are updated, and the diversity of the population is lost. Researchers used variation-perturbation strategies to increase population diversity to obtain more search information. For example, Guo et al.^22^ integrated the follower position update mechanism in Sparrow search algorithm to perturb the algorithm, and combined Cauchy-Gauss variation strategy to help the algorithm jump out of the local optimal solution. Pan et al.^23^ introduced adaptive Gauss-Cauchy hybrid mutation perturbation to enhance the ability of dung beetle algorithm to coordinate its local development and global exploration. Wang et al.^24^ proposed a decreasing control strategy for the convergence factor of disturbance index to achieve a good coordination between the exploration and development capabilities of Gray Wolf algorithm. Chen et al.^25^ designed a perturbation strategy for wavelet optimal solutions to improve population diversity and avoid the algorithm falling into local optimality. Vu-Huu et al.^26^ proposed a push-process technique to improve the effectiveness of the BA algorithm by reducing the wide distribution of the optimal global solution of its to produce an intervention in the BA algorithm, so as to achieve a true global optimal that can be exposed in several generations without many computational times. In this paper, PID control is designed to disturb the optimal solution to get new individuals, so that individuals in the population can be optimized in multiple directions, increase the diversity of the population and improve the search ability of the algorithm.

PID algorithm has excellent performance in the field of control, combining proportional control, integral control and differential control, aiming at fast and stable output of setpoint^27^. In the fitting regression problem, the algorithm is optimized according to the value of the adaptation function. Therefore, the optimal individual is fine-tuned by PID controlling the optimal fitness function value, which helps the algorithm to jump out of the local extreme value and avoid premature maturity. The mathematical expression of PID control is:

9 $$ u(k) = K_{p} err(k) + \frac{{K_{p} T}}{{T_{i} }}\sum\limits_{n = 0}^{k} {err(n) + } \frac{{K_{p} T_{d} }}{T}(err(k) - err(k - 1)) $$

In the formula,$K_{p}$ is the proportionality constant,$K_{i} = (K_{p} *T)/T_{i}$ is the integral constant, $K_{d} = (K_{p} *T_{d} )/T$ is a microconstant. The experiment $K_{p} = 0.4$ has obtained a good result.

#### Curve adaptive weight factor

In order to better coordinate the global search and local exploration capabilities, and enhance the optimization and later development capabilities of the algorithm iteration, Cuong-Le et al.^28^ proposed a new cuckoo search algorithm (NMS-CS) based on the Levy flight, which uses the Levy distribution to calculate the random step size, and randomly selects the newly created functions (convex function, concave function, linear function, etc.) to control the parameters in the CS algorithm, so as to expand the search space in the early stage of algorithm iteration and improve the development ability in the late iteration stage. Inspired by the idea of inertial weighting in the improved particle swarm optimization^29^, this paper adds curve adaptive weights to the update formula (2) of the rolling ball dung beetle, and the weight factor is guided by the cosine function, and with the increase of the number of iterations, the curve changes similar to the cosine function (0-π range), so the weight factor keeps decreasing slowly in the early stage of iteration, the decline rate accelerates in the middle of iteration, and slowly decreases again in the late iteration, which can ensure that the algorithm slows down the global search performance in the early stage and improves the local optimal in the later stage. The expression is,

10 $$ w_{1} = (\cos (\pi \cdot t/T_{\max } ) + w_{\max } )(w_{\max } + w_{\min } )/2 + a $$

In the formula, *t* is the current number of iterations, $T_{\max }$ is the maximum number of iterations, *a* is the adjustment factor,$w_{\max }$ and $w_{\min }$ are the maximum and minimum values of the factor, respectively. In formula (2), the adaptive weight of the curve is added and modified as follows:

11 $$ x_{i} (t + 1) = w_{1} \times x_{i} (t) + \tan \theta \left| {(1 - w_{1} ) \times x_{i} (t) - x_{t} (t - 1)} \right| $$

**Algorithm 3:**
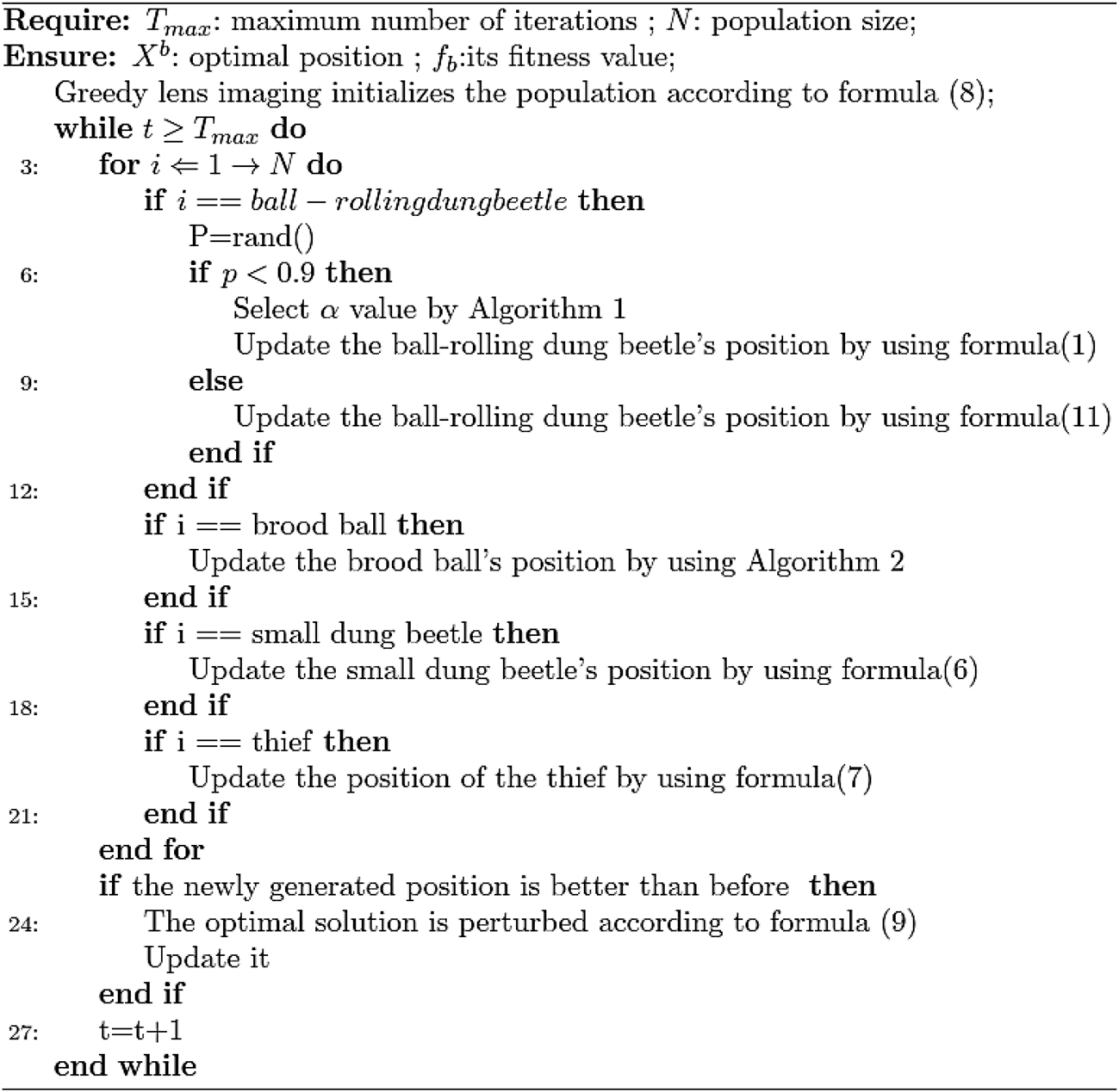
IDBO pseudo-code

### Function optimization simulation experiment and result analysis

#### Simulation experiment

In order to test the optimization performance of IDBO algorithm, 9 benchmark test functions in CEC2005 dataset^30^ are selected in this paper, as shown in Table 1. There are 4 categories: unimodal problem $f_{1} - f_{4}$, Basic multimodal problem $f_{{_{7} }} ,f_{{_{10} }}$, Extended multimodal and hybrid composite problems $f_{13} ,f_{15} ,f_{20}$.

**Table 1:** Benchmark functions.

Function	D	Search space	$f_{\min }$
$f_{1} (x) = \sum\limits_{i = 1}^{n} {x_{i}^{2} }$	30	$[ - 100,100]^{D}$	0
$f_{2} (x) = \sum\limits_{i = 1}^{n} {\left| {x_{i} } \right| + \prod\limits_{i = 1}^{n} {\left| {x_{i} } \right|} }$	30	$\left[ { - 10,10} \right]^{D}$	0
$f_{3} (x) = \sum\limits_{i = 1}^{n} {(\sum\limits_{j - 1}^{i} {x_{j} )^{2} } }$	30	$\left[ { - 100,100} \right]^{D}$	0
$f_{4} (x) = \max_{i} \{ \left| {x_{i} } \right|,1 \le i \le n\}$	30	$\left[ { - 100,100} \right]^{D}$	0
$f_{7} (x) = \sum\limits_{i = 1}^{n} {ix_{i}^{4} + random[0,1)}$	30	$\left[ { - 1.28,1.28} \right]^{D}$	0
$f_{10} (x) = - 20\exp ( - 0.2\sqrt {\frac{1}{n}\sum\limits_{i = 1}^{n} {x_{i}^{2} } } ) - \exp (\frac{1}{n}\sum\limits_{i = 1}^{n} {\cos (2\pi x_{i} )) + 20 + e}$	30	$\left[ { - 32,32} \right]^{D}$	0
$$\begin{gathered} f_{13} (x) = \sum\limits_{i = 1}^{n} {u(x_{i} ,5,100,4)} + \hfill \\ 0.1\{ \sin^{2} (3\pi x_{1} ) + \sum\limits_{i = 1}^{n - 1} {(x_{i} - 1)^{2} [1 + \sin^{2} (3\pi x_{i + 1} )] + (x_{n} - 1)^{2} [1 + \sin^{2} (2\pi x_{n} )]\} } \hfill \\ \end{gathered}$$	30	$\left[ { - 50,50} \right]^{D}$	0
$f_{15} (x) = \sum\limits_{i = 1}^{11} {\left[ {a_{i} - \frac{{x_{i} (b_{i}^{2} + b_{i} x_{2} )}}{{b_{i}^{2} + b_{i} x_{3} + x_{4} }}} \right]}^{2}$	4	$\left[ { - 5,5} \right]^{D}$	0.0003
$f_{20} (x) = - \sum\limits_{i = 1}^{4} {c_{i} \exp ( - \sum\limits_{j = 1}^{6} {a_{ij} (x_{j} - p_{ij} )^{2} )} }$	6	$\left[ {0,1} \right]^{D}$	− 3.3219

#### Analysis of algorithm test results

**Figure 1:**
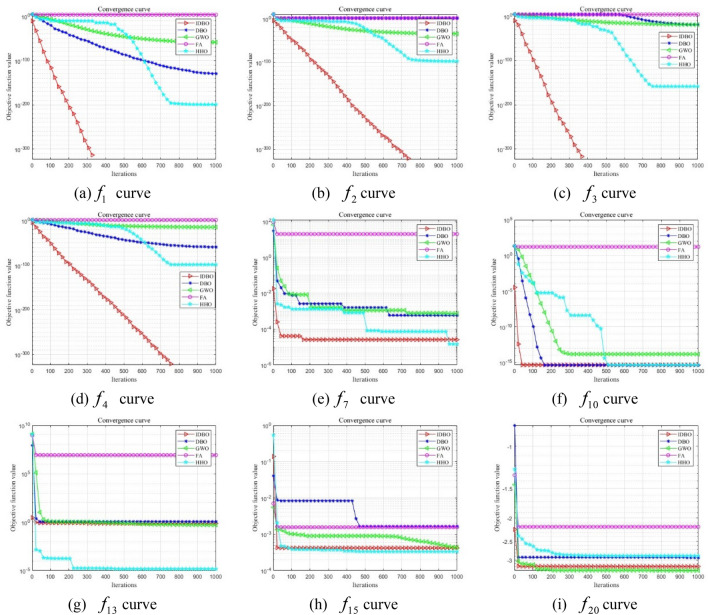
Convergence curve of the test function.

IDBO algorithm is compared with standard Dung Beetle algorithm (DBO), Grey Wolf algorithm (GWO), Firefly algorithm (FA) and Harris Eagle algorithm (HHO) to optimize test functions. In order to reduce the chance of the experiment, set the same experimental parameters, The population size of each algorithm *N* = 30, maximum number of iterations $T_{\max }$ = 1000. Perform 30 independent experiments on 9 test functions respectively. Figure 1 draws the fitness iteration convergence comparison curve, and evaluates and compares it by convergence speed and iteration number. The experimental results are shown in Table 2.

**Table 2:** Comparison of test function results.

		IDBO	DBO	HHO	GWO	FA
$f_{1}$	Best	0	0	4.5E−221	1.5E−148	93.89529
	Worse	0	1.4E−215	1E−192	2.2E−142	2378.495
	Std	0	0	0	5.6E−143	631.0331
	Avg	0	4.5E−217	3.4E−194	2.3E−143	665.1144
$f_{2}$	Best	0	3.3E−160	1E−115	1.37E−84	1.580033
	Worse	0	1.9E−97	7.7E−97	1.69E−79	10.562
	Std	0	3.47E−98	1.41E−97	3.15E−80	2.098315
	Avg	0	6.3E−99	2.57E−98	1.06E−80	5.926974
$f_{3}$	Best	0	1.4E−277	8.6E−203	1.29E−76	225.8074
	Worse	0	1.9E−162	1.3E−163	7.1E−63	1491.548
	Std	0	0	0	1.3E−63	318.7335
	Avg	0	6.3E−164	4.4E−165	2.37E−64	689.5716
$f_{4}$	Best	0	6.3E−152	7.2E−107	4.74E−49	2.741784
	Worse	0	5.9E−108	2.58E−93	8.44E−45	20.19865
	Std	0	1.1E−108	4.71E−94	1.71E−45	5.200433
	Avg	0	2E−109	8.6E−95	8.15E−46	10.32102
$f_{7}$	Best	5.42E−06	4.86E−05	4.74E−07	3.05E−05	0.016502
	Worse	0.00012	0.001928	0.000172	0.000636	0.29265
	Std	2.37E−05	0.000422	4.03E−05	0.000153	0.079691
	Avg	2.9E−05	0.000577	3.73E−05	0.000221	0.10475
$f_{10}$	Best	4.44E−16	4.44E−16	4.44E−16	4E−15	3.43768
	Worse	4.44E−16	4.44E−16	4.44E−16	7.55E−15	13.618
	Std	0	0	0	6.49E−16	2.628233
	Avg	4.44E−16	4.44E−16	4.44E−16	4.12E−15	9.729815
$f_{13}$	Best	0.059958	1.35E−32	4.59E−09	1.81E−07	1.421995
	Worse	0.511519	0.097371	9.58E−05	0.101882	740,146
	Std	0.099024	0.017857	1.8E−05	0.03048	134,630.9
	Avg	0.207361	0.003978	8.87E−06	0.009989	34,123.03
$f_{15}$	Best	0.000338	0.000307	0.000308	0.000307	0.0005
	Worse	0.017615	0.001271	0.001227	0.020363	0.001699
	Std	0.001615	0.000705	0.00038	0.006385	0.000942
	Avg	0.000745	0.000713	0.000316	0.000307	0.000843
$f_{20}$	Best	− 3.22025	− 3.322	− 3.31926	− 3.32199	− 3.18287
	Worse	− 1.82072	− 3.1327	− 3.09772	− 3.13448	− 1.56463
	Std	0.359287	0.071029	0.071884	0.065279	0.508591
	Avg	− 2.82475	− 3.25912	− 3.2005	− 3.26297	− 2.56725

CEC2005 test set, $f_{1} - f_{4}$ function structure is relatively simple, test algorithm convergence performance; The $f_{{_{7} }} ,f_{{_{10} }}$ function has a local optimal solution and tests the algorithm's ability to balance global development and local exploration in the search space. Function $f_{13} ,f_{15}$ and $f_{20}$ tests the ability of the algorithm to deal with mixed complex problems. From Fig. 1(a–d), it can be clearly seen that IDBO algorithm converges first and has a faster convergence speed as the number of iterations increases. It shows that the introduction of greedy lens imaging reverse learning to initialize the population can effectively improve the population quality and accelerate the convergence speed. From Fig. 1e, f, IDBO can quickly jump out of the local extreme solution at the early stage of iteration, so as to achieve the global optimal explanation, and the introduction of curve adaptive weight factor and PID control optimal solution perturbation strategy can help the algorithm get rid of local extrema and enhance the global optimization ability. As can be seen from Fig. 1g–i, IDBO also has excellent optimization ability in dealing with mixed complex problems. It can be seen that the improved strategy of dung beetle optimization algorithm is effective.

In Table 2, the IDBO algorithm and the evaluation indexes of the four algorithms are the best value, the worst value, the standard deviation and the average value respectively. By observing the optimization results in Table 2, we can see that except for $f_{7} ,f_{10}$ and $f_{13}$, IDBO can find theoretical optimal values in other benchmark functions. Standard DBO can find the theoretical optimal value of the $f_{1}$ function, the other algorithms failed to find the theoretical optimal value. IDBO, standard DBO, and HHO have the same standard deviation on the $f_{1} ,f_{3}$ function, the mean and standard deviation of IDBO optimization results on unimodal function are 0. The comprehensive performance of IDBO is obviously better than DBO, HHO, GWO and FA in terms of optimization accuracy and stability. For multi-modal functions, the optimization results of $f_{7}$ function show that IDBO and HHO have similar standard deviations and average values, and their orders of magnitude are 10^–5^, respectively, while DBO, GWO and FA are 10^–4^, 10^–4^ and 10^–2^, respectively. The optimization results of $f_{10}$ function show that IDBO, DBO and HHO have the same standard deviation and average value, which are 0 and 4.44E−16 respectively. The index values of GWO and FA are not good. In conclusion, IDBO is significantly better than the comparison algorithm in handling the balance between local exploration and global development. For the mixed composite function, the optimization results of $f_{13}$ function show that the optimal value of DBO is 10^–32^, while IDBO is not good. The optimization results of function $f_{15}$ and $f_{20}$ show that the optimal values of the five algorithms are close to the theoretical optimal values, indicating that IDBO, DBO, HHO, GWO and FA algorithms have the ability to deal with this complex problem.

## Model principle and parameter optimization process

### Long short-term memory regression prediction model

**Figure 2:**
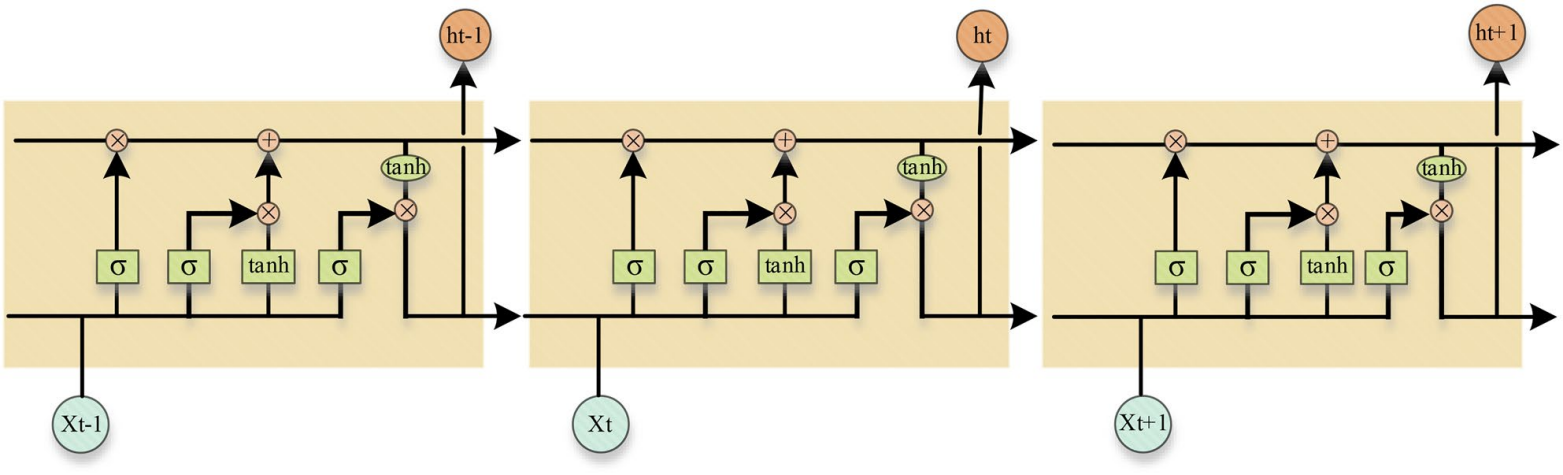
LSTM cell structure.

Long short-term memery (LSTM) is an improvement of recurrent neural networks (RNN), widely used in machine translation^31^, speech recognition^32^, and image description. The LSTM network structure newly establishes a memory unit with feedback connections in the direction of time, which is reflected in the addition of three gate structures, namely, forget gate, input gate and output gate. At time step *t*, input data is $x_{t}$, Then the hidden state of the previous moment is $h_{t - 1}$, and the cell state is $c_{t - 1}$. The LSTM model structure is shown in Fig. 2.

Forgetting gate $f_{t}$, control the forgetting degree of the unit state at the previous moment:

12 $$ f_{t} = \sigma (W_{f} x_{t} + U_{f} h_{t - 1} + b_{f} ) $$

In the formula, $W_{f}$ is the weight matrix of the input $x_{t}$,$U_{f}$ is the weight matrix of the hidden state $h_{t - 1}$ at the previous time, $b_{f}$ is the biased variable, $\sigma$ is the sigmoid function.

Enter gate $i_{t}$ to control the input of new information:

13 $$ i_{t} = \sigma (W_{i} x_{t} + U_{i} h_{t - 1} + b_{i} ) $$

In the formula, $W_{i}$ is the weight matrix of the input $x_{t}$,$U_{i}$ is the weight matrix of the hidden state $h_{t - 1}$ at the previous time, $b_{i}$ is the biased variable, $\sigma$ is the sigmoid function.

Output gate A, control output degree:

14 $$ o_{t} = \sigma (W_{o} x_{t} + U_{o} h_{t - 1} + b_{o} ) $$

In the formula, $W_{o}$ is the weight matrix of the input $x_{t}$,$U_{o}$ is the weight matrix of the hidden state $h_{t - 1}$ at the previous time, $b_{o}$ is the biased variable, $\sigma$ is the sigmoid function.

New cell status $\tilde{c}_{t}$, update the current cell status:

15 $$ \tilde{c}_{t} = \tanh (W_{c} x_{t} + U_{c} h_{t - 1} + b_{c} ) $$

In the formula, $W_{c}$ is the weight matrix of the input $x_{t}$,$U_{c}$ is the weight matrix of the hidden state $h_{t - 1}$ at the previous time, $b_{c}$ is the biased variable,$\tanh$ is a hyperbolic tangent function.

Calculate the cell state A at the current moment and update the hidden state S:

16 $$ \begin{gathered} c_{t} = f_{t} \odot c_{t - 1} + i_{t} \odot \tilde{c}_{t} \hfill \\ h_{t} = o_{t} \odot \tanh (c_{t} ) \hfill \\ \end{gathered} $$

In the formula,$\odot$ stands for element-by-element product.

### Parameter optimization based on IDBO

The objective of IDBO algorithm is to help LSTM model select appropriate parameters, which are LSTM hidden unit number, maximum training period, initial learning rate and L2 regularization parameter. So the fitness function of dung beetles is defined as,

17 $$ fitness = \frac{1}{N}\sum\limits_{i = 1}^{N} {\left( {y_{i} - \hat{y}_{i} } \right)} $$

In the formula, *N* is the number of samples, $\hat{y}_{i}$ is the predicted value for sample *i*, $y_{i}$ is the actual value for sample *i*.

**Figure 3:**
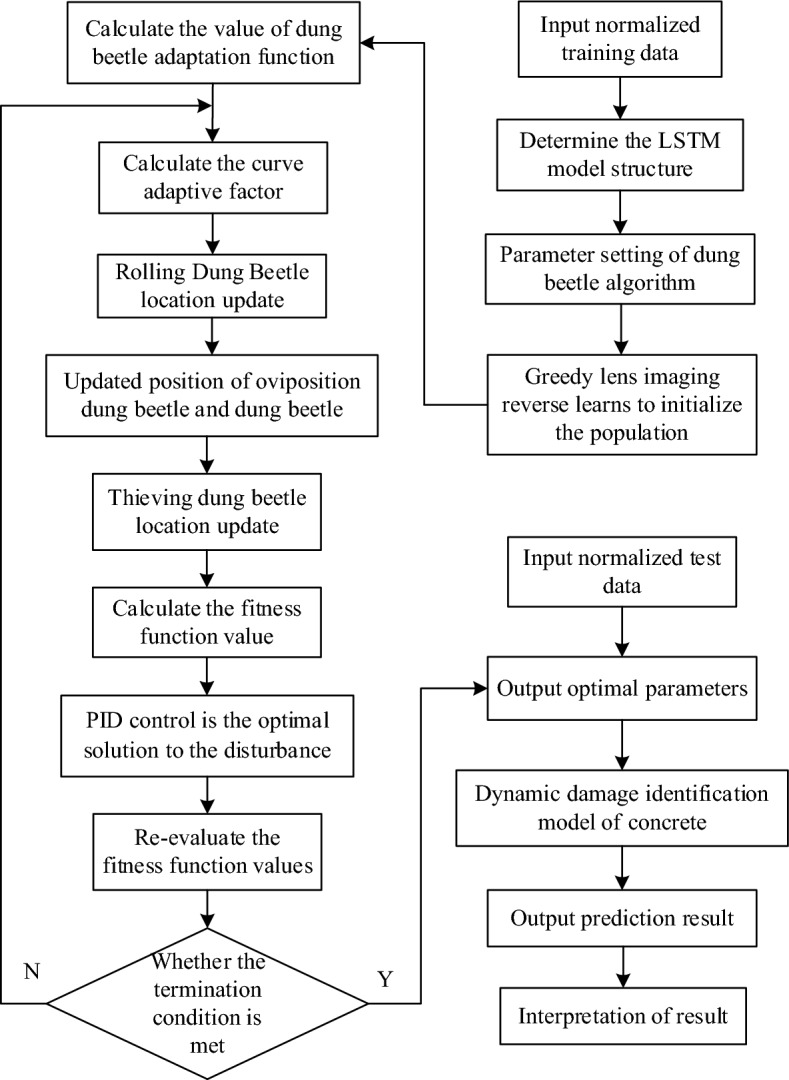
Flow chart of IDBO optimizing LSTM parameters.

Set the total number of dung beetles SearchAgent-n = 30, among them, the proportion of rolling dung beetles, laying dung beetles, small dung beetles and thieving dung beetles was 20%, 40%, 20% and 20% respectively. The maximum number of iterations Max_iter = 10, the number of variables dim = 4, and the search range of variables are determined. The parameter optimization process of LSTM model based on IDBO algorithm is shown in Fig. 3.

## Example analysis

### Data sources

The data comes from a series of experiments designed by our group, and the concrete with a wide range of strength grades C40 is selected as the experimental specimen, and the newly constructed model is verified by using this experimental data.

#### Concrete specimen preparation

**Figure 4:**
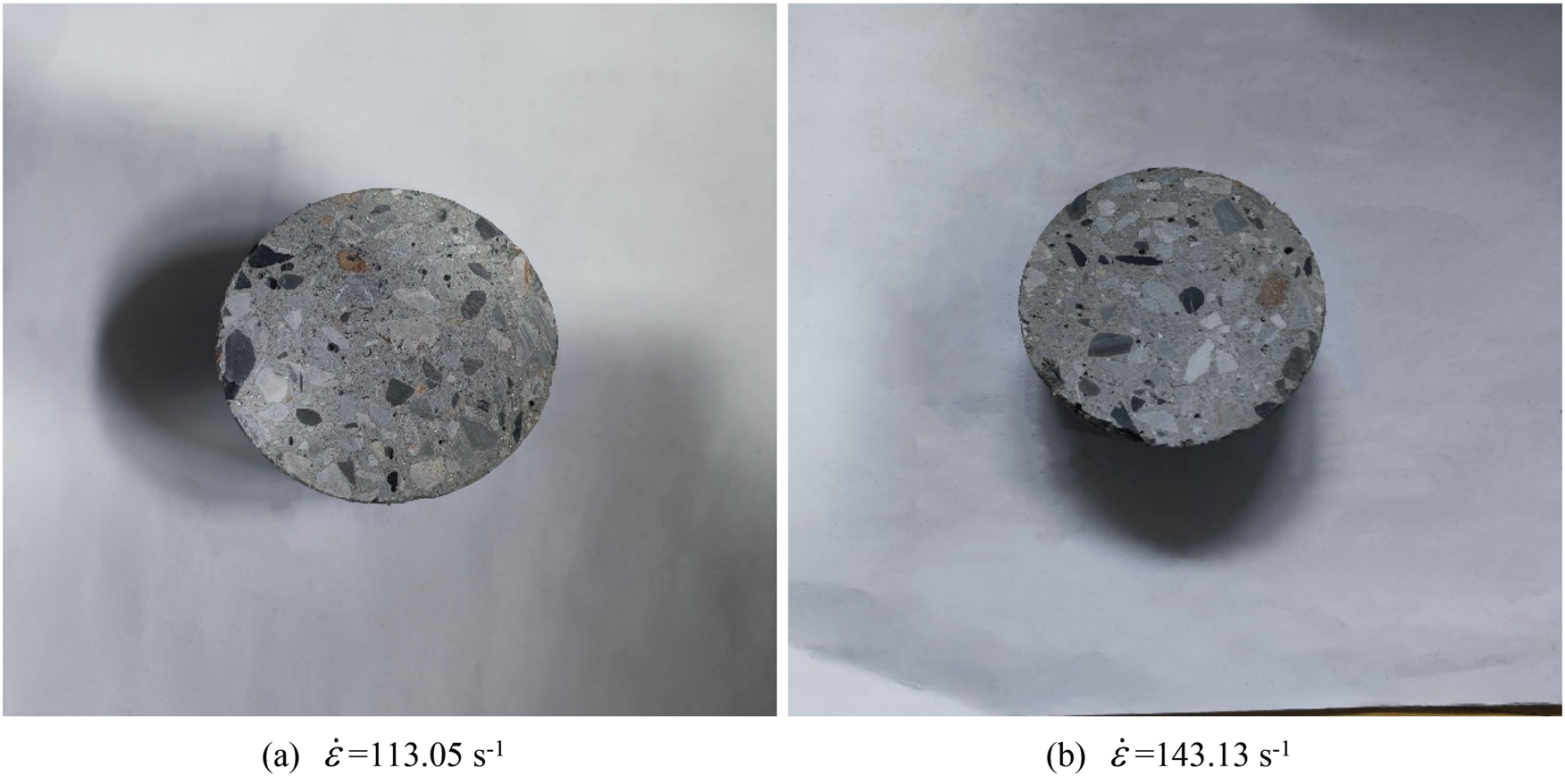
Concrete sample.

The test specimen is a cylinder with a diameter of 70 mm and a height of 35 mm. The cement in the concrete material composition is ordinary Portland cement with a strength grade of 42.5, the coarse aggregate is made of pebbles and gravel with a particle size of 5–10 mm, and the fine aggregate is made of medium coarse river sand, with a large particle size of 5 mm, a fineness modulus of 2.8–3.0, and a mud content of less than 1%. The admixture is polycarboxylic acid high-efficiency superplasticizer mother liquor. The mix ratio of steel fiber concrete specimens is: cement: 425 kg/m^3^, sand: 600 kg/m^3^, stone: 1132 kg/m^3^, water: 184 kg/m^3^, water reducer: 8 kg/m^3^, Steel fiber: 39 kg/m^3^. The concrete specimen is shown in Fig. 4.

#### Experimental results of separated Hopkinson bar (SHPB)

**Figure 5:**
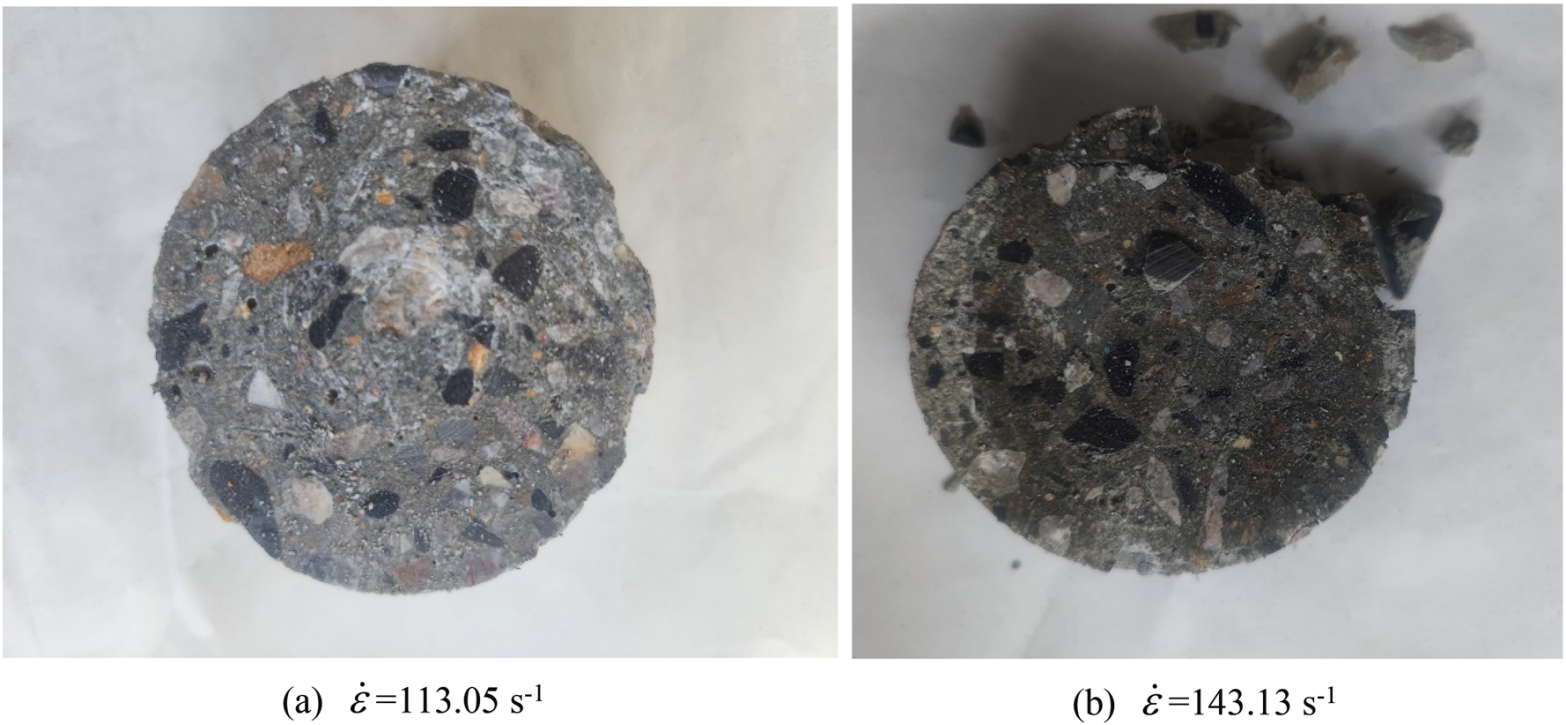
Concrete specimen after loading.

In order to verify the accuracy of the test results, we performed 6–10 repeated tests for each strain rate at room temperature, and took the average of the multiple tests as the test results for the loading condition. The concrete specimen after loading is shown in Fig. 5.

### recessive modulus constitutive equation of concrete material

The dynamic failure of materials is a process in which different forms of micro-damage (micro-cracks, micro-voids, micro-shear bands, etc.) accumulate at a finite rate over time. The macroscopic continuous damage *D* is defined as follows:

18 $$ D = \frac{{\sigma_{0} - \sigma }}{{\sigma_{0} }} $$

In the formula, $\sigma_{0}$ is non-damaging material stress, $\sigma$ is the apparent stress of the damaged material.

In general, material damage evolves with the rheological process, so damage *D* is $\varepsilon$ function of strain $\varepsilon$. However, A large number of dynamic tests show that the evolution of material damage under impact load depends on both strain and strain rate, i.e.$D = D\left( {\varepsilon ,\dot{\varepsilon }} \right)$. From a macroscopic point of view, from the perspective of systems science, the constitutive relation is equivalent to the relationship between the cause (input) and the effect (output) of a system, that is, the system identification problem. Therefore, the one-dimensional constitutive relationship of steel fiber reinforced concrete under different strain rates can be expressed as,

19 $$ \sigma \left( t \right) = f\left[ {\varepsilon \left( t \right),\dot{\varepsilon }\left( t \right)} \right]\varepsilon \le \varepsilon_{th} $$

20 $$ \sigma \left( t \right) = f\left[ {\varepsilon \left( t \right),\dot{\varepsilon }\left( t \right),D\left( t \right)} \right] = f\left[ {\varepsilon \left( t \right),\dot{\varepsilon }\left( t \right),t^{ - 1} \left( D \right)} \right] \varepsilon > \varepsilon_{th} $$

In the formula, $\varepsilon_{th}$ is the damage threshold (0.75 times the peak strain), measured by the "damage freezing method". The damage value *D* cannot be directly determined in the test. Considering that the damage value *D* is a function of time, the inverse function of time with respect to the damage value *D* is taken as the damage.

### Experimental parameter settings

See Table 3.

**Table 3:** The main parameters of the algorithm.

Algorithm	Parameter
LSTM	Initial learning rate: 0.005, Maximum number of iterations: 200, Number of implicit units:200, Descending factor = 0.2
GWO	Linear weight: *ω* = 2 − *t* *(2/*T*_*max*_)
DBO	Light source coefficient: *b* = 0.3, Disturbance coefficient: *k* = 0.1
IDBO	Scale Factor: *k* = 10,000, Weight Factor: *ω*_*min*_ = 0.2, *ω*_*max*_ = 0.9, Adjustment Factor: *a* = 0.45, Proportionality Constant: *k*_*p*_ = 0.4

### Analysis of simulation results

**Figure 6:**
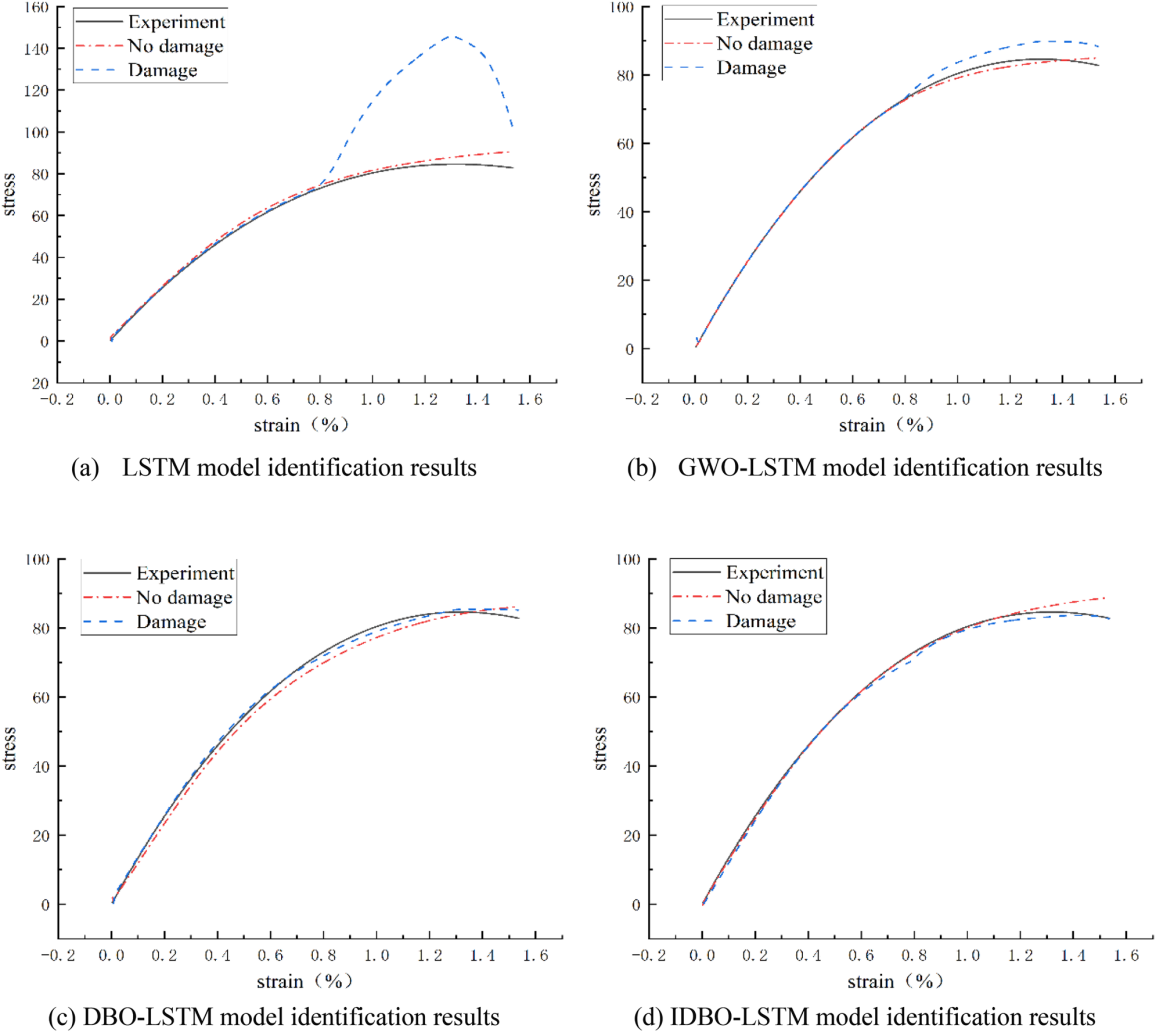
Comparison of model identification results. Annotation*: the damage-free curve (red dashed line) is obtained by using strain and strain rate as inputs and stress as output. The damage curve (blue line) is obtained by using strain, strain rate, and time as inputs, and stress as output.

In order to verify the effectiveness of the proposed concrete dynamic constitutive identification model, a steel fiber reinforced concrete specimen with a strain rate of 113.05 s^−1^ is taken as an example. The experimental data were input into the trained LSTM, DBO-LSTM, GWO-LSTM and IDBO-LSTM models for identification, and the identification results of the four models are shown in Fig. 6.

As can be seen from Fig. 6, the LSTM model has the lowest recognition ability among the four constitutive recognition models. LSTM can define macroscopic continuous damage. However, after taking into account the damage data, the curve predicted by the LSTM model does not agree with the test curve, so it cannot be verified to be accurate in defining the damage. Figure 6b, c show that the LSTM model optimized with GWO or DBO has improved the accuracy of its definition of damage, and the verification ability of DBO optimization is relatively high. In Fig. 6e, within the scope of $\varepsilon \le \varepsilon_{th}$,the IDBO-LSTM prediction curve is in good agreement with the test curve, but after the deformation of the concrete specimen exceeds the limit of model learning, the test curve and the prediction curve deviate, and we believe that it is the occurrence of damage that causes the deviation of the curve, as shown in Fig. 6d red dotted line. After considering the damage evolution, that is, adding time as the inverse function of damage, the prediction curve of the IDBO-LSTM model is in good agreement with the test curve in the whole strain range, as shown in Fig. 6d blue underlined.

**Figure 7:**
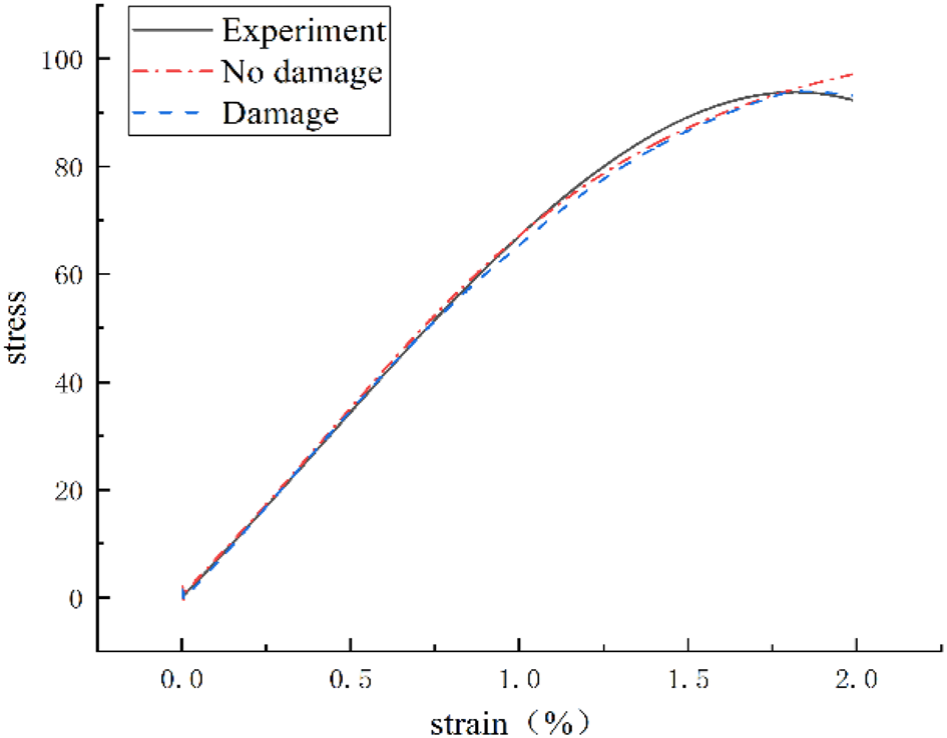
Identification results of specimens with a strain rate of 143.13 s^−1^.

In order to further verify the universality of the IDBO-LSTM model, a steel fiber reinforced concrete specimen with a strain rate of 143.13 s^−1^ was selected, and the sample test data was input into the IDBO-LSTM model, and the identification results are shown in Fig. 7.

**Figure 8:**
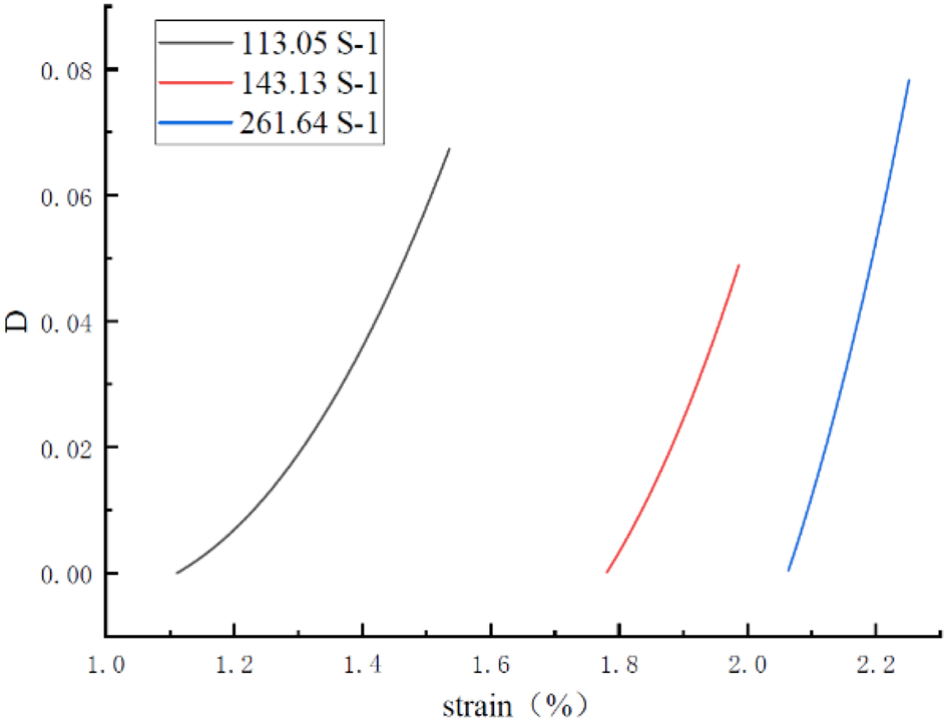
Damage evolution curves.

From the above identification, the continuous damage is determined as a function of strain and strain rate according to Eq. (18), in the form of the evolution of damage D with strain for different constant strain rates as shown in Fig. 8.

## Conclusions

In this paper, a concrete dynamic constitutive identification model (IDBO-LSTM identification model) based on improved dung beetle algorithm and optimized long short-term memory neural network is proposed. Based on the thermal activation damage evolution model, the damage and rheology are separated by the apparent stress–strain curve of concrete with damage evolution, and the LSTM method is used to model the system. The greedy lens imaging reverse learning strategy, curve adaptive weight factor and PID control optimal solution disturbance strategy are introduced to improve the original shortcomings of the Dung Beetle algorithm, and the improved Dung Beetle algorithm is combined with LSTM method to identify the dynamic constitutive of concrete, and the conclusions are as follows:

- The greedy lens imaging reverse learning strategy was introduced to initialize the population, which improved the uneven position of the initial dung beetles, and the greedy idea was used to greatly reduce the optimization time in the algorithm iteration process, improve the quality of the initial population, and increase the diversity of the population.
- The combination of curve adaptive weight factor and PID control optimal solution disturbance strategy dynamically adjusts the balance between global development and local exploration of the algorithm, helps the algorithm quickly jump out of the local optimal value, and improves the optimization accuracy and stability of the algorithm.
- By combining IDBO-LSTM neural network technology with SHPB test, the constitutive response and damage evolution rate of concrete under impact load can be identified by different input and output modes according to the test data without any constitutive assumptions in advance.

### Ethical and informed consent for data used

The data supporting the findings of this study are available within the supplementary materials.

### Supplementary Information

Supplementary Information.
